# Cytogenetic analysis of quinoa chromosomes using nanoscale imaging and spectroscopy techniques

**DOI:** 10.1186/1556-276X-8-463

**Published:** 2013-11-06

**Authors:** Zhong Yangquanwei, Suresh Neethirajan, Chithra Karunakaran

**Affiliations:** 1 BioNano Laboratory, Biological Engineering, University of Guelph, Guelph, Ontario, N1G 2 W1, Canada; 2 Canadian Light Source, Saskatoon, Saskatchewan, S7N 2 V3, Canada

**Keywords:** Chromosomes, Synchrotron radiation, Soft X-ray spectromicroscopy, Image analysis, Atomic force microscopy, Cytogenetics, DNA-protein composition

## Abstract

Here we present a high-resolution chromosomal spectral map derived from synchrotron-based soft X-ray spectromicroscopy applied to quinoa species. The label-free characterization of quinoa metaphase chromosomes shows that it consists of organized substructures of DNA-protein complex. The analysis of spectra of chromosomes using the scanning transmission X-ray microscope (STXM) and its superposition of the pattern with the atomic force microscopy (AFM) and scanning electron microscopy (SEM) images proves that it is possible to precisely locate the gene loci and the DNA packaging inside the chromosomes. STXM has been successfully used to distinguish and quantify the DNA and protein components inside the quinoa chromosomes by visualizing the interphase at up to 30-nm spatial resolution. Our study represents the successful attempt of non-intrusive interrogation and integrating imaging techniques of chromosomes using synchrotron STXM and AFM techniques. The methodology developed for 3-D imaging of chromosomes with chemical specificity and temporal resolution will allow the nanoscale imaging tools to emerge from scientific research and development into broad practical applications such as gene loci tools and biomarker libraries.

## Background

Function of the genome depends on the chromosome architecture [1]. For predictive gene diagnosis and for personalized medicine, simultaneous understanding of the structural and chemical makeup of chromosomes is essential [2]. To integrate biomolecular and clinical data for cancer research, spectral-based biomarker libraries of chromosomes of species are required.

Conventional cytogenetic analysis such as karyotyping involves the observation of defects on the surface of chromosomes using optical microscopy and thereby relates to the physiological attributes and disease state of the species. Classical banding methods provide only basic information regarding the structure and identities of chromosomes, while spectral karyotyping has the potential to provide improved characterization of aberrant chromosomes that contain DNA sequences [3]. Recently, the fluorescence *in situ* hybridization (FISH) technique has been commonly adopted [4] as a sensitive tool for determining aberrations on chromosomes. A major drawback of the FISH technique is that the fluorescence intensity only roughly reflects the local density of packed DNA inside chromosomes and does not correspond to the topographic height [5,6]. In addition, higher cost, staining, and the long analysis protocol make the FISH technique cumbersome, expensive, less accurate, and manual.

Internal interphase chromosome architecture and composition have not been addressed thoroughly because of the lack of visualization tools. There is a dire need for rapid real-time high-throughput genomic mapping and molecular marker identification tool for isolation of quantitative trait loci, and thereby designing crops with stress, insect, and drought tolerance [7]. Nanoscale imaging techniques allow us to examine the ultrastructure of cells in a detailed fashion [8]. Accurate topology of the chromatin (DNA and protein composition) network inside a single chromosome has not yet been characterized precisely. A chromosome is made up of DNA and associated proteins and other compounds in the nanoscale domain containing the genomic information. To understand the structure–property relationship of any organic material, quantitative compositional analysis at length scales below 100 nm is required [9].

Synchrotron-based nanoscale imaging tools offer the possibility to understand the embedding of the chromatin interaction networks inside the chromosomes. Advances in nanoscale imaging techniques especially synchrotron-based radiation enable the molecular cytogenetics for accurate visualization and analysis of chromosomes at molecular resolution. Specifically, soft X-ray spectromicroscopy is well suited for analyzing the spatial distribution of specific elements in unstained wet or dry biological specimens [10-12]. The synchrotron-based scanning transmission X-ray microscopy (STXM) technique provides quantitative chemical mapping at a spatial resolution of 25 to 30 nm.

Genomic resources on the minor crops are less investigated. In contemporary times, quinoa has become highly appreciated for its nutritional value, as its protein content is very high (14% by mass) [13]. However, relatively little is known about quinoa cytogenetics beyond the species’ chromosome number (*n* = 36). To unlock the potential of rapid cytogenetic analysis, nanoscale imaging is essential in the single-molecule characterization of chromosome architecture.

Soft X-ray absorption spectroscopy using STXM at the nitrogen or carbon edge is sensitive to differentiate DNA and protein [11,12], and can be used for chemical mapping of chromosomes. It is a challenge to acquire precise details on the chromosome due to fixation of the chromosomes with agents such as formaldehyde or osmium tetroxide leading to radiation damage and shrinkage. In our study, we precisely characterized the composition of quinoa chromosomes by exposing only 1 ms of dwell time to avoid the radiation damage. Here we have shown for the first time the advantages of utilizing atomic force microscopy (AFM) and scanning electron microscopy (SEM) for the morphological characterization (at the atomic and nanoscale level) and STXM for the compositional characterization (at the nanoscale level) of chromosomes. The morphology and the biochemical properties inside a single quinoa chromosome were determined by utilizing nanoscale imaging tools such as STXM, AFM, SEM, and confocal laser scanning microscopy (CLSM).

## Methods

### Root tip preparation

Chromosomes were isolated from the meristematic tissue of quinoa root tips. Seeds of *Chenopodium quinoa* were germinated on moist filter papers in petri dishes at room temperature in the dark over 48 h. For cytogenetic analysis, primary root tips were pretreated with 2 mM 8-hydroxyquinoline for 4 h at room temperature, followed by incubation in ice-cold water overnight, fixed in methanol-glacial acetic acid (3:1 ratio), and stored at -4°C for further use.

### Cell suspension

About 2-mm meristematic tips from each root were removed followed by dissection into the smallest possible sections. The root tip sections were macerated in a 200-μL enzyme reaction mixture for 4 h at 37°C. After the incubation time, the solution was filtered through a 50-μm gauze twice. To this filtered solution, 2 ml of 75 mM KCl solution was added. This suspension was centrifuged for 70 min at 20°C at 760 rpm. The supernatant was discarded and the precipitate was re-suspended in 3 ml of the 3:1 fixative (methanol: acetic acid) and again centrifuged for 7 min at 760 rpm/75 g at 20°C. The above process was repeated five times. After discarding the supernatant from the final wash, the resulting pellet was re-suspended in 200 μL of the 3:1 fixative.

### AFM imaging

In an attempt to prepare a full set of chromosomes, the samples were prepared not from the cell suspension but using the maceration technique reported by Neethirajan et al. [14]. Briefly, the pretreated quinoa root tips were incubated in an enzyme solution of 2% cellulase, 2% pectolyase, and 1.5% macerozyme for 90 min at 37°C, followed by squashing on the glass slides by tapping with the tip of forceps in 30% acetic acid. The squashed specimens were further cleaned using 1X SSC to remove the cellular debris, before being imaged using AFM.

The samples were first observed with an inverted phase contrast optical microscope (Nikon Eclipse Ti, Nikon Instruments, Tokyo, Japan) and photographed to determine the location of the chromosomes to be studied by AFM. The glass slides were marked underneath as a possible region of interest for AFM imaging. AFM (Agilent AFM/SPM 5500, Agilent Technologies, Chandler, AZ, USA) was used for scanning the chromosome samples. Standard silicon cantilevers with a spring constant of 48 N m^-1^ were used. All AFM measurements were carried out in atmospheric air at room temperature of approximately 25°C using the intermittent contact mode with resonant frequency of around 190 kHz. The scan speeds were in the range of 0.2 to 0.3 Hz. Both topographic and error signal images were acquired simultaneously during AFM imaging. The same cantilever tip was used for imaging all the chromosomes to avoid difference in tip profiles. The analysis and measurement of the images were made using SPIP software (Image Metrology, Copenhagen, Denmark).

### SEM imaging

Twenty microliters of cell suspension in 3:1 fixative was dropped from a height of 60 cm onto an ice-cold moistened glass slide. Just as the fixative evaporates, one drop of 45% acetic acid was applied to the area of the dropped cell suspension. A cover slide was immediately applied, and the whole slide was laid, coverslip-side down, on dry ice. After 15 min, the coverslip was pried off, and the glass slide was immediately immersed in a fixative solution of 2.5% glutaraldehyde in 75 mM cacodylate buffer and dried using the critical point drying method. SEM images were collected using Hitachi S-570 SEM (Tokyo, Japan) using Quartz PCI software (Quartz Imaging Corp., Vancouver, Canada).

### STXM imaging and spectroscopy

About 2 μl of the cell solution was casted on the Si_3_Ni_4_ membrane window (approximately 75-nm thick and 0.5 × 0.5 mm^2^ area, Norcada Inc., Edmonton, Canada) and air dried. The samples were then stained using the nucleic acid stain, SYTO-9 (Invitrogen Canada, Burlington, Canada). The stained samples were observed using a MRC 1024 confocal laser scanning microscope (CLSM, Bio-Rad, Hemel Hempstead, UK), and individual chromosome locations were identified prior to X-ray imaging. The SYTO 9 stain used for confocal microscopy does not affect the spectral signatures collected using STXM as the concentration was quite low. The staining is not essential for the STXM study but helps to identify chromosomes from other plants much faster. The Si_3_Ni_4_window with the samples was then mounted on the STXM sample holder and imaged using the STXM at the soft X-ray spectromicroscopy beamline of the Canadian Lights Source Inc. in transmission mode using a phosphor-PMT detector [15,16]. The X-ray energies at the C1s region (280 to 320 eV) were used to confirm the chromosomes and to determine its composition at a spatial resolution of 25 nm. All data were analyzed using the aXis2000 program (http://unicorn.mcmaster.ca/aXis2000.html). All transmission data were converted to optical densities (absorption) using the incident flux on the sample by a recording spectrum where there was no sample on the Si_3_Ni_4_ window.

In STXM, X-ray images were recorded at the specific absorption edges (287.4 eV for DNA and 288.2 eV for protein) to confirm the presence of chromosomes and a series of images at different X-ray energies called as stack for quantification of DNA and protein from individual chromosomes. The stack data were first aligned using the Zimba procedure [17] which uses the cross correlation of successive images. The reference spectra of protein and DNA [18] were then normalized to an absorbance of 1 nm of material using the theoretical absorption calculated using the composition and density [19]. The stack data of chromosomes were then converted into individual component maps (thickness in nanometers) using the single value decomposition (SVD) method that uses the linear regression fitting of the reference spectra.

## Results and discussion

Classical banding protocols for studying chromosomes provide only the basic morphological information regarding the structures of chromosomes, while spectral karyotyping using nanoscale imaging techniques is chromosome specific and provides additional chemical information and improved characterization of aberrant chromosomes that contain DNA sequences not identifiable using conventional banding methods.

The chromosome number of *Chenopodium quinoa* is 2*n* = 4*X* = 36 with a diploid genome of 967 Mbp, but the chromosome sizes are very small and basically without distinguishing parameters to be able to enable traditional karyotyping or develop biomarker libraries. Our optimized protocol helped to successfully isolate chromosomes from the quinoa root tip and was able to image without staining using SEM, AFM, STXM, and CLSM.

**Figure 1:**
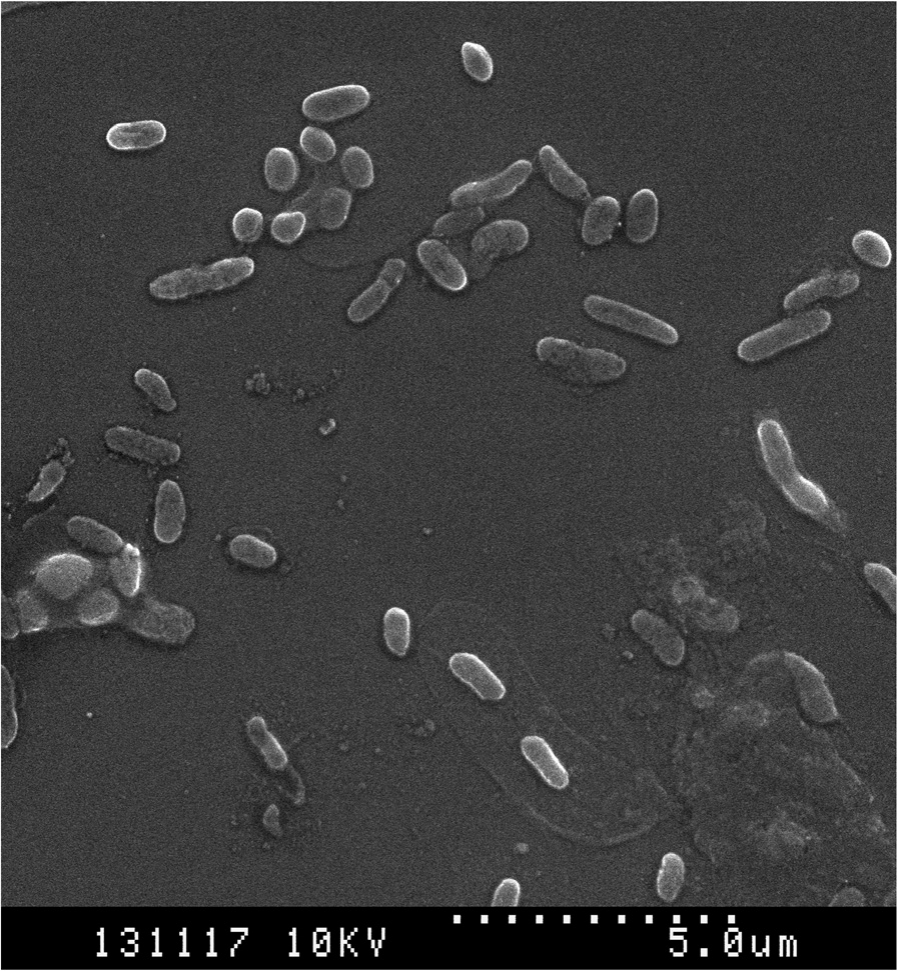
**Air-dried processed scanning electron microscopy image of quinoa chromosomes.** The chromosomes appear uniformly dense with scarcely distinguishing parameters. The centromere is barely visible. Scale bar, 5 μm.

**Figure 2:**
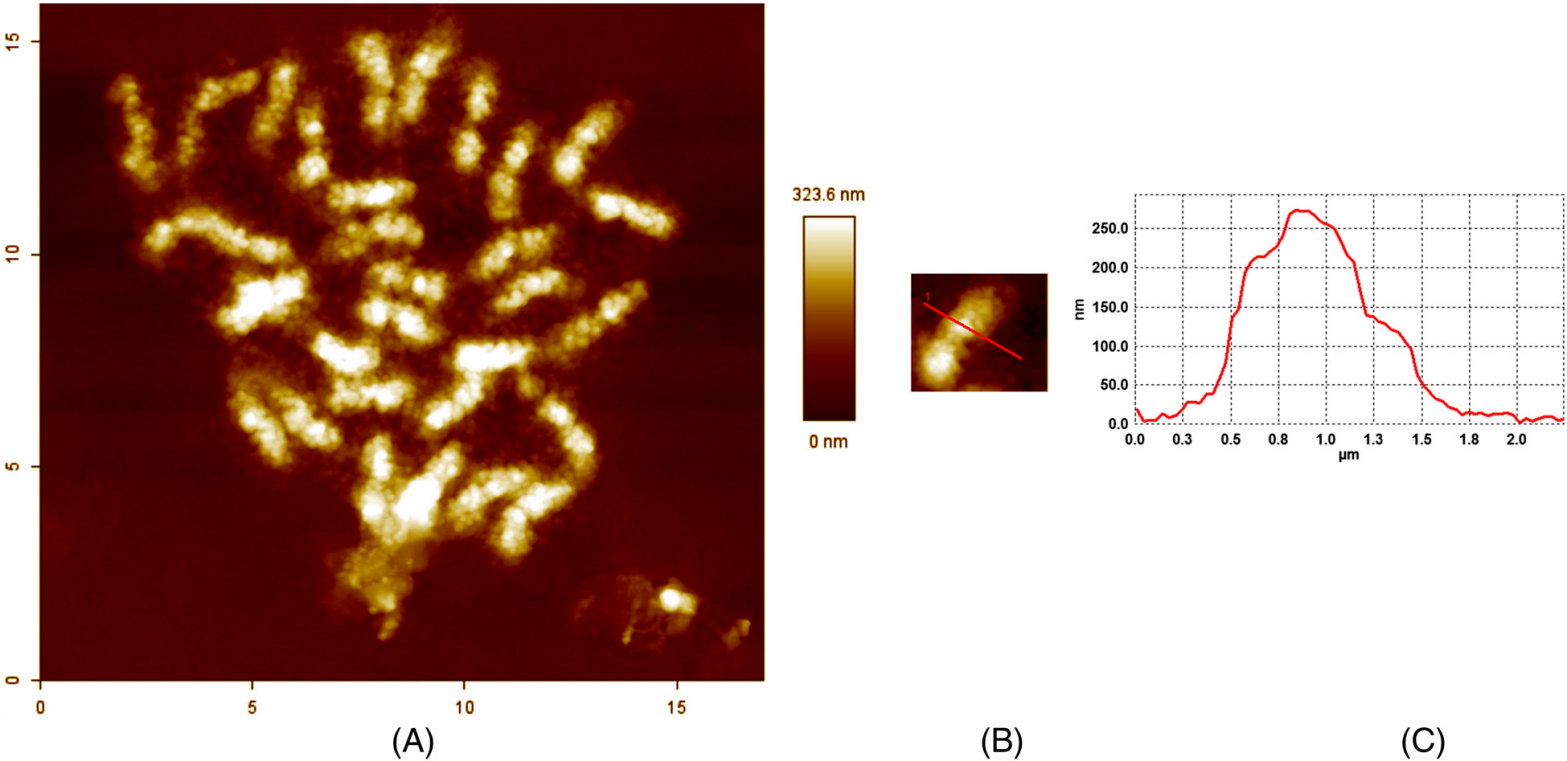
**Topography, surface analysis, and section profile. (A)** The topography was recorded in air using intermittent contact mode AFM. The topography exhibits a vertical brightness range of 300 nm. The chromatid appears divided by grooves in subunits corresponding to the units visible in SEM. The surface morphology corresponds to the SEM image. **(B)** Surface analysis of the quinoa chromosome by AFM. **(C)** Section profile of the chromosome along the line in **(B)**.

The SEM (Figure 1) and AFM (Figure 2) images of quinoa chromosomes showed a preserved cylindrical morphology with length ranging between 600 and 3,100 nm. A total of 32 chromosomes are visible as a set using AFM, out of which two pairs of chromosomes with secondary constriction are distinguished. Out of 36, only 32 chromosomes are being observed (Figure 2A) in the AFM image mainly due to the smaller size of chromosomes not facilitating the analysis and possibly due to chromosome rearrangements. The quinoa chromosome as imaged using AFM appears 'mushy’ and is smaller than normal-sized chromosomes of other species. The length of chromosomes ranges between 600 nm to 3.1 μm. A region of interest was selected to provide the cross-sectional profile of the quinoa chromosomes. The thickness of quinoa chromosomes as observed through a typical cross-section profile of AFM imaging shows that the chromosome thickness is not uniform and varies between 160 to 310 nm (Figure 2B). This indicates the occurrence of condensation of chromatin fiber in the early metaphase stage.

After the confirmation of the presence of chromosomes in the silicon window using video microscopy, a series of STXM X-ray images were recorded at X-ray energies from 280 to 300 eV (stacks) to quantify the distribution of DNA and protein from each chromosome. The stacks were first aligned using a cross-correlation procedure and then converted into optical densities.

**Figure 3:**
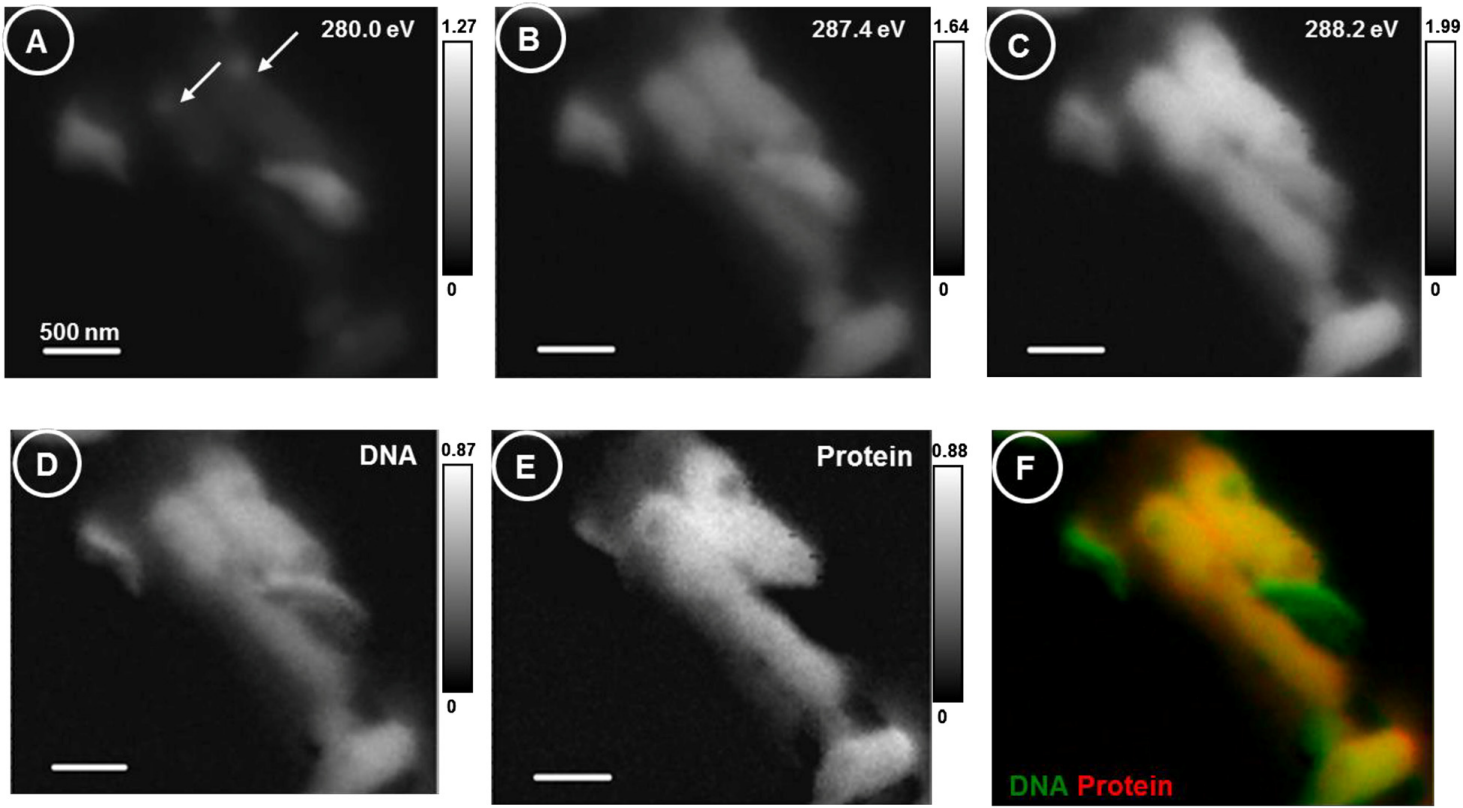
**STXM X-ray absorption images recorded to map the distribution of DNA and protein on chromosomes. (A)** Pre-edge at 280.0 eV. **(B)** DNA absorption at 287.4 eV. **(C)** Protein absorption at 288.2 eV. **(D)** Distribution of DNA (B - A). **(E)** Distribution of protein [C - (B + A)]. **(F)** Composite image showing distribution of DNA and protein. All scale bars are in optical density.

Figure 3 shows the X-ray images recorded at the absorption edges of DNA and protein and shows the DNA-protein distribution of a group of chromosomes using STXM. The X-ray images recorded at the specific absorption energy of DNA or protein were used to identify the chromosomes from a larger area (to differentiate them from other plant debris) as well for the quick mapping on the spatial distributions of the components. The pre-edge image at 280.0 eV shows non-carbonaceous spots on three chromosomes, indicating the presence of phosphorus and other differences in DNA composition between chromosomes. If the density of DNA and protein is assumed as 1.0 g/cm^3^, the optimal thickness of the sample required for STXM for good (30%) transmission through the sample is less than 200 nm. The thickness of quinoa chromosomes being larger than 200 nm did not facilitate ideal penetration for the X-ray imaging. The STXM image displays the chromosome to be a dense X-ray structure.

**Figure 4:**
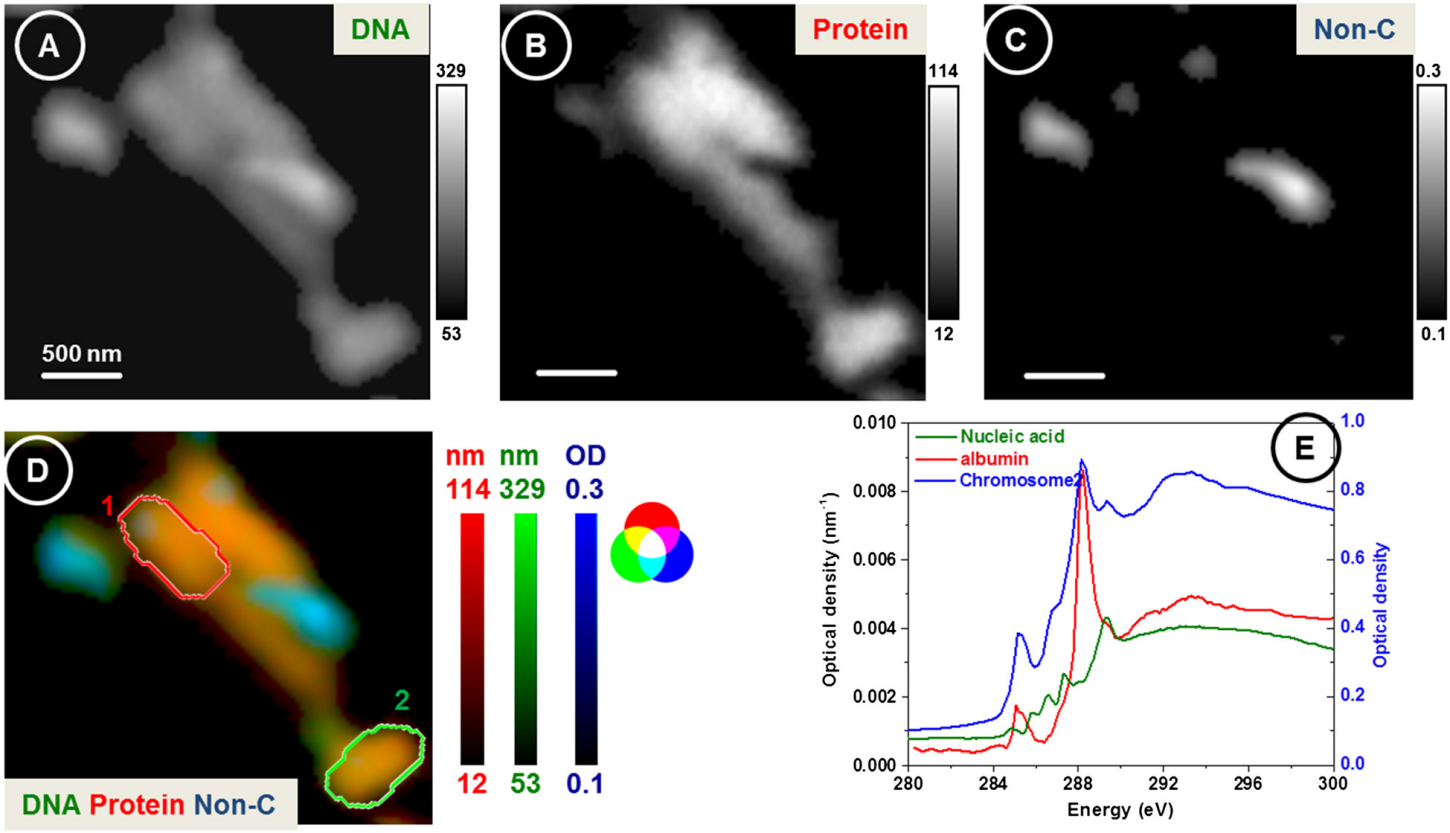
**Compositional maps of chromosomes. (A)** DNA. **(B)** Protein. **(C)** Non-carbonaceous compounds. **(D)** Composite image. **(E)** Absorbance reference spectra of 1 nm of albumin and nucleic acid. The scale bars of **(A)** and **(B)** are in nanometers and **(C)** is in optical density. Two chromosomes are marked in red (1) and green (2) for comparison.

The analysis of the detailed energy map fitted with reference spectra of DNA and protein using STXM (Figure 4) reveals that the quinoa chromosome is primarily composed of DNA and protein, with some non-carbon components present inside and outside the chromosomes (X-ray image recorded at 280.0 eV). Proteins from plants and animals do not have differences in the spectral signatures due to the large number of amino acids present. The reference spectra of protein (albumin) and DNA (nucleic acid) normalized to an absorbance of 1 nm of material using the theoretical absorption using the composition and density are shown in Figure 4. The stack data of chromosomes were then converted into individual component maps (thickness or scale bar in nanometers) using the SVD method that uses the linear regression fitting of the reference spectra.

Figure 4 shows the distribution of DNA and protein (in nanometers) in different chromosomes. The reference spectra of albumin and nucleic acids have strong transition peaks at 288.2 and 289.3 eV that can be attributed to the C1s → 1π* C = O of carbonyl bond of the amide group from the protein and C1s → 1π* C = N of DNA bases, respectively. It can be shown that the spectra extracted from chromosome 2 have an optical density below 1.0 which shows that the spectra are not saturated due to the thickness of the chromosomes, and hence, STXM data can be used for quantitative measurements. The compositional maps or images (Figure 4) show that DNA is present in higher amounts than protein in each chromosome. The relative amounts of DNA and protein at any location in a chromosome can be determined by extracting the spectra from a specific location and fitting with the reference spectra. In addition, the size, shape, and total amounts of DNA and protein can also be determined from the STXM data. For example, two similar chromosomes were manually segmented as shown in Figure 4 and compared for their size and composition (Figure 4, Table 1). Although the shape and area of the two chromosomes are similar, the total DNA and protein between the two chromosomes differ (Table 1).

**Table 1:** Comparison of morphological and compositional characteristics of two chromosomes

**Name**	**Area (μm**^**2**^**)**	**DNA (nm)**	**Protein (nm)**
Chromosome 1	0.32	123 ± 46.5	68.3 ± 28.1
Chromosome 2	0.29	111 ± 55.8	55.8 ± 29.1

The integration of the image data from chromosomal morphologies from AFM and SEM, and the chemical mapping from STXM allowed visualization and identification of the quinoa chromosomes. The morphological and biochemical analysis on chromosomes using the STXM provided the local chemical architecture of the quinoa metaphase chromosomes.

Our results demonstrates that AFM in combination with STXM could serve as a valuable tool for extracting spatiotemporal information from intra- and interphase chromosomes Superimposition of the topographical image from AFM and the STXM images provides precise analysis of the fine structural and chemical makeup of the chromosomes.

The enormous amount of genetic information inside the chromosome is accessible only under *in vivo* conditions via loops during mitosis until maximum condensation of the metaphase stage [20]. Unlike the staining-based FISH technique or CLSM or SEM techniques, STXM and AFM offer imaging of the chromosomes under *in vivo* conditions. The advantages of STXM include less radiation damage to the chromosomes compared to electron microscopy and without alteration of chemical specificity due to the stains. In addition, the possibility of precisely estimating the composition of chromosomes using 3-D spectromicroscopy technique makes STXM an attractive tool [21].

AFM while complementing STXM can detect close proximity of the genomic loci in the interphase chromosomes through 3-D mapping of chromosomes and can pinpoint DNA and protein interactions in loci on the same (cis) as well as on different chromosome (trans). The prospect of imaging the single chromosome at the nanoscale level will aid not only the direct visualization but also spatial characterization of the configuration of genes within the chromatin. The advantages of label-free imaging of chromosomes using STXM includes avoiding of the concerns such as non-uniform binding of labeling agents and photo-bleaching.

## Conclusions

The result of this study bridges the methodological gap between the chromosome banding and molecular biology techniques for genetic diagnostics through single-molecule characterization and biochemical label-free imaging of chromosome architecture at subcellular resolution. The methodology developed in this study demonstrates the potential of developing precise nanoscale spectral karyotypes of plant species chromosomes and establishing a map of genome attributing regions (quantitative trait loci) for measuring morphological phenotypes. Nanoscale imaging-assisted cytogenetic analysis will aid in understanding the pathomechanism of disease of crops and in complementing the marker-assisted breeding through identification of genetic linkage maps. Precise molecular markers have the ability for influencing high-throughput genome sequencing and the characterization of the genetic diversity for the crop species. The agricultural biotechnology market currently lacks efficient tools or systems for conducting studies to understand the genome biology focusing on chromosomal and DNA structural variations. The results of this study have the potential to develop a new class of technology suitable for rapid and on-field disease detection of crops.

## Competing interests

The authors declare that they have no competing interests.

## Authors’ contributions

The manuscript was written through contributions of all authors. All authors have given approval to the final version of the manuscript.

## References

[B1] Van Steensel B Dekker J Genomics tools for unraveling chromosome architecture Nat Biotechnol 2010 10 1089 1095 20944601 10.1038/nbt.1680 PMC3023824

[B2] Collins FS Green ED Guttmacher AE Guyer MS A vision for the future of genomics research. US National Human Genome Research Institute Nature 2003 422 835 847 10.1038/nature01626 12695777

[B3] Padilla-Nash HM Barenboim-Stapleton L Difilippantonio MJ Ried T Spectral karyotyping analysis of human and mouse chromosomes Nat Protoc 2006 6 3129 3142 17406576 10.1038/nprot.2006.358 PMC4772431

[B4] Jehan Z Uddin S Al-Kuraya KS In-situ hybridization as a molecular tool in cancer diagnosis and treatment Curr Med Chem 2012 22 3730 3738 22680920 10.2174/092986712801661031

[B5] Di Bucchianico S Giardi MF de Marco P Ottaviano L Botti D Atomic force microscope nanolithography on chromosomes to generate single-cell genetic probes J Mol Recognit 2011 24 608 618 10.1002/jmr.1094 21472812

[B6] Yoshino T Sugiyama S Hagiwara S Fukushi D Shichiri M Nakao H Kim JM Hirose T Muramatsu H Ohtani T Nanoscale imaging of chromosomes and DNA by scanning near-field optical/atomic force microscopy Ultramicroscopy 2003 97 81 87 10.1016/S0304-3991(03)00032-9 12801660

[B7] Mochida K Shinozaki K Advances in omics and bioinformatics tools for systems analyses of plant functions Plant Cell Physiol 2011 12 2017 2038 22156726 10.1093/pcp/pcr153 PMC3233218

[B8] Hummel E Guttmann P Werner S Tarek B Schneider G Kunz M Frangakis AS Westermann B 3D ultrastructural organization of whole *Chlamydomonas reinhardtii* cells studies by nanoscale soft X-ray tomography PLoS One 2012 12 e53293 23300909 10.1371/journal.pone.0053293 PMC3534036

[B9] Ade H Stoll H Near-edge x-ray absorption fine-structure microscopy of organic and magnetic materials Nat Mater 2009 8 281 290 10.1038/nmat2399 19308087

[B10] Ogura T Direct observation of unstained wet biological samples by scanning-electron generation X-ray microscopy Biochem Biophys Res Commun 2010 391 198 202 10.1016/j.bbrc.2009.11.031 19900411

[B11] Ade H Zhang X Cameron S Costello C Kirz J Williams S Chemical contrast in X-ray microscopy and spatially resolved XANES spectroscopy of organic specimens Science 1992 258 972 975 10.1126/science.1439809 1439809

[B12] Williams S Zhang X Jacobsen C Kirz J Lindaas S Van't Hof J Lamm SS Measurements of wet metaphase chromosomes in the scanning transmission X-ray microscope J Microsc 1993 2 155 165

[B13] Kumpun S Maria A Crouzet S Evrard-Todeschi N Girault J Lafont R Ecdysteroids from *Chenopodium quinoa* wild., an ancient andrean crop of high nutritional value Food Chem 2011 4 1226 1234

[B14] Neethirajan S Hirose T Wakayama J Tsukamoto K Kanahara H Sugiyama S Karyotype analysis of buckwheat using atomic force microscopy Microsc Microanal 2011 4 572 577 21749742 10.1017/S1431927611000481

[B15] Kaznatcheev KV Karunakaran C Lanke UD Urquahart SG Obst M Hitchcock AP Soft X-ray spectromicroscopy beamline at the CLS: commissioning results Nucl Instrum Methods Phys Res, Sect A 2007 582 96 99 10.1016/j.nima.2007.08.083

[B16] Fakra S Kilcoyne ALD Tyliszczak T Scintillator detectors for scanning transmission X-ray microscopes at the advanced light source Eighth International Conference on Synchrotron Radiation Instrumentation, 705, 973–906 2004 San Francisco, California, USA: The American Institute of Physics

[B17] Jacobsen C Wirick S Flynn G Zimba C Soft x-ray spectroscopy from image sequences with sub-100 nm spatial resolution J Microsc 2000 197 2 173 184 10.1046/j.1365-2818.2000.00640.x 11543408

[B18] Koprinarov IN Hitckcock AP McCrory CT Childs RF Quantitative mapping of structured polymeric systems using singular value decomposition analysis of soft x-ray images J Phys Chem B 2002 21 5358 5364

[B19] Lawrence JR Swerhone GDW Leppard GG Araki T Zhang X West MM Hitchcock AP Scanning transmission X-ray, laser scanning, and transmission electron microscopy mapping of the exopolymeric matrix of microbial biofilms App Env Microbio 2003 69 5543 5554 10.1128/AEM.69.9.5543-5554.2003 PMC194976 12957944

[B20] Wanner G Formanek H A new chromosome model J Struct Biol 2000 2 147 161 11162737 10.1006/jsbi.2000.4310

[B21] Wang J Hitchcock AP Karunakaran C Prange A Franz B Harkness T Lu Y Obst M Hormes J 3D chemical and elemental imaging by STXM spectro-tomography, XRM2010 AIP Conf Proc 2010 1365 215 218

